# Glucagon-like peptide-1 attenuated carboxymethyl lysine induced neuronal apoptosis via peroxisome proliferation activated receptor-γ

**DOI:** 10.18632/aging.203351

**Published:** 2021-07-29

**Authors:** Haoqiang Zhang, Bing Song, Wenwen Zhu, Lili Liu, Xiqiao He, Zheng Wang, Ke An, Wuyou Cao, Jijing Shi, Shaohua Wang

**Affiliations:** 1 Department of Endocrinology, Affiliated Zhongda Hospital of Southeast University, Nanjing 210000, Jiangsu Province, China; 2 School of Medicine, Southeast University, Nanjing 210000, Jiangsu Province, China; 3 Department of Endocrinology, First Affiliated Hospital of Jinzhou Medical University, Jinzhou 1210001, Liaoning Province, China

**Keywords:** carboxymethyl-lysine, apoptosis, glucagon-like peptide-1, peroxisome proliferator activated receptor-γ

## Abstract

Backgrounds and aims: The role of peroxisome proliferator activated receptor-γ (PPAR-γ) in neuronal apoptosis remains unclear. We aim to investigate the role of PPAR-γ in glucagon-like peptide-1 (GLP-1) alleviated neuronal apoptosis induced by carboxymethyl-lysine (CML).

Materials and Methods: *In vitro*, PC12 cells were treated by CML/GLP-1. Moreover. the function of PPAR-γ was blocked by GW9662. *In vivo*, streptozotocin (STZ) was used to induce diabetic rats with neuronal apoptosis. The cognitive function of rats was observed by Morris water maze. Apoptosis was detected by TUNEL assay. Bcl2, Bax, PPAR-γ and receptor of GLP-1 (GLP-1R) were measured by western blotting or immunofluorescence.

Results: *In vitro* experiment, CML triggered apoptosis, down-regulated GLP-1R and PPAR-γ. Moreover, GLP-1 not only alleviated the apoptosis, but also increased levels of PPAR-γ. GW9662 abolished the neuroprotective effect of GLP-1 on PC12 cells from apoptosis. Furthermore, GLP-1R promoter sequences were detected in the PPAR-γ antibody pulled mixture. GPL-1 levels decreased, while CML levels increased in diabetic rats, compared with control rats. Additionally, we observed elevated bax, decreased bcl2, GLP-1R and PPAR-γ in diabetic rats.

Conclusions: GLP-1 could attenuate neuronal apoptosis induced by CML. Additionally, PPAR-γ involves in this process.

## INTRODUCTION

Prevalence of type 2 diabetes (T2DM) [1–4] and Alzheimer's Disease (AD) [5, 6] had been a challenge in China and worldwide. T2DM is involved in the progress of AD via several mechanisms, including amyloid-β accumulation [7, 8], hyperphosphorylation of tau protein [9, 10] and oxidative stress [11] result in neuronal apoptosis [12]. Advanced glycation end products (AGEs), formed by non-enzymatic-reaction in the condition of chronic hyperglycemia, are involved in various diabetic complications, including degenerative neuropathy. Indeed, many studies suggested that AGEs promoted the damage of neurons by binding to receptor of advanced glycation end products (RAGE). Chen et al [13] showed high glucose induced apoptosis by PC12 cells *in vitro* and STZ induced hyperphosphorylation of tau protein by rats *in vivo*. Additionally, recent study [12] indicated that induced neuronal apoptosis in SH-SY5Y cells. However, the mechanism of AGEs involves in neuronal apoptosis needs to be further studied.

GLP-1 is a kind of incretin mainly secreted by intestinal L cells, open-type intestinal epithelial endocrine cells located in the distal ileum and colon [14]. It could bind to GLP-1R, and then, maintain plasma glucose stabilization via the balance of insulin and glucagon [15]. GLP-1R is a member of G protein-coupled receptor [16], expression in the heart, pancreatic, stomach, intestine, lung, kidney, gut nerve and brain [17]. Wei et al reported the expression of GLP-1R in brain firstly and indicated that GLP-1R in brain, heart and pancreatic have the same deduced amino acid sequences [18]. Although most GLP-1 was secreted by L cells is inactivated soon (within 2 minutes) by dipeptidyl peptidase 4 (DPP-4), study showed that microglial cells [19] as well as a cluster of neurons in the nucleus of the solitary tract and a smaller number of neurons that extend laterally from the nucleus of the solitary tract [20] could secrete GLP-1. This kind of GLP-1 may bind to the GLP-1R in the brain, and then trigger neuroprotective effect.

Recent studies showed neuroprotective effect of GLP-1 (or GLP-1 analogue) and its receptor [21, 22]. Kappe et al found GLP-1 decreased in ob/ob mice with cognition declines [19]. Moreover, Liraglutide administration could improve the cognitive function of mice [23, 24]. GLP-1R-deficient mice have a phenotype characterized by a learning deficit that is restored after hippocampus GLP-1R gene transfers. However, rats over-expressing GLP-1R in the hippocampus show improved learning and memory [25]. Additionally, GLP-1 attenuates AGEs-induced RAGE up-regulation in SH-SY5Y cells and improved the AGEs-induced cells vitality declines [12].

PPAR-γ is not only a potential target to treat diabetes by alleviating insulin resistance [26], but also play a promising role in neuroprotective effect [27, 28]. In recent studies, researchers demonstrated that PPAR-γ may contribute to prevent apoptosis from AGEs and oxidative stress [29, 30]. However, the role of PPAR-γ in the process of RAGE medicated neuronal apoptosis is still unclear. In another work, PPAR-γ was up-regulated by the administration of GLP-1 [31, 32]. But, thus far, the relationship between GLP-1R and PPAR-γ remains need to be further explored in PC12 cells.

CML, is one of the most studied components of AGEs [33]. It could induce damage and apoptosis of podocyte [34], foam cells [35] and neurons [36]. In addition, GLP-1 showed its anti-apoptosis in many different cell types [37]. Although, PPAR-γ, associated with apoptosis [29], is regulated by GLP-1 [31], the mechanism of GLP-1 alleviated apoptosis of PC12 cells induced by CML is still unclear. So, we designed this study to explore the role of PPAR-γ in GLP-1 alleviated apoptosis induced by CML in PC12 cells.

## MATERIALS AND METHODS

### Cell culture and differentiation

PC12 cell line was purchased from American Type Culture Collection and stimulated to form a neuron-like phenotype [38, 39] with nerve growth factor (Novoprotein, Shanghai, China, Catalogue No.: C060) (50ng/ml) for 3 days. And then, they were cultured in Dulbecco’s modified eagle’s medium (DMEM) (Gibco by Thermo Fisher Scientific TM, Suchoo, China) supplemented with 5% fetal bovine serum (FBS) (Gibco, Australia, Catalogue No.: A3161001C), 1% penicillin/streptomycin (Beyotime, Shanghai, China, Catalogue No.: C0222) and 10% horse serum (Beyotime, Shanghai, China, Catalogue No.: C0262). Cells were harvested for passaging when plates were 90% confluent.

### CML stimulating, GLP-1 treatment and GW9662 blocking assay

For CML (MyBioSource, San Diego, USA, Catalogue No.: MBS390113) stimulating, cells (5×103) in 100 μl DMEM were added to 96-well plates with different concentrations of CML (0, 12.5, 25, 50, 75, and 100μg/ml) for 6, 12 and 24 hours. GLP-1 (Bioss, Beijing, China, Catalogue No.: bs-0038P) treatment was performed with GLP-1 at concentrations of 0nM, 50nM, 100nM, 200nM, and 500nM in the medium with cells stimulated by 50 μg/ml CML for 24 hours. GW9662 (MedChemExpress LLC, Shanghai, China, Catalogue No.: 22978-25-2) blocking administration was conducted by 0, 1, 5, 10, 20, and 50 μM GW9662 in PC12 cells with 50 μg/ml CML and 100nM GLP-1 for 24 hours. After CML stimulating, GLP-1 treatment and GW9662 blocking assay, CCK-8 assay was performed according to the protocol of manufacturer (Jiangsu KeyGEN BioTECH Corp., Ltd, Nanjing, China, Catalogue No.: KGA317-2).

### Animal housing and treatment

Adult male Wistar rats (6 weeks old) were purchased from Beijing HFK Biotechnology Co., Ltd. (Beijing, China) and housed in a specific pathogen-free animal center. All rats were fed with their specific diet and bacteria free water. To get hyperglycemia models, they were induced by STZ (Sigma-Aldrich, Saint Louis, MO, USA, Catalogue No.: V900890) (a single dose intraperitoneal 60mg/kg) prepared in in a 0.1 M citrate buffer (pH 4.5) after 12 hours fasting. After 72 hours injections, fasting blood glucose >16.7mmol/L was admitted as diabetes. 8 diabetic rats and 8 control rats were divided into diabetic rat group and control rat group respectively. This present study was approved by the Animal Studies Committee of our institution and conducted in accordance with the Guide for the Care and Use of Laboratory Animals.

### Morris water maze

After a period of 8 weeks from the success of the diabetic models, Morris water maze tests were conducted to detect the cognitive function. Before training, rats were allowed to swim freely to adapt the environment for 1 min without the platform. Then, each rat was trained for five consecutive days to find the platform. The time of each rat took to find the platform and the total length of the path were recorded as escape latency and path length respectively. Additionally, we also recorded the percentage of time spent in the target quadrant as well as the frequency of crossing the platform area.

### TUNEL assay

TUNEL kit (Servicebio, Wuhan, China, Catalogue No.: G1507-50T) were used to measure levels of apoptosis according to the manufacturer's protocol.

### Western blotting

After CCK-8 test, CML (50μg/ml) was selected to induce PC12 cells apoptosis for 24 hours. Additionally, GLP-1 (100nM) was used to treatment CML induced cells damage. Moreover, GW9662 at a concentration of 5μM was administrated to inhibit PPAR-γ. Western blotting was carried out according to a previously described protocol [40]. Briefly, total proteins from tissue or cells were extracted using radioimmunoprecipitation (RIPA) (Wanlei Biotechnology Co. Ltd, Shenyang, China, Catalogue No.: WLA016a) with 1% phenylmethanesulfonyl fluoride added. Subsequently, a BCA protein assays kit (Wanlei Biotechnology Co. Ltd, Shenyang, China, Catalogue No.: WLA004b) was used to measure protein concentration according to the manufacturer's protocol. Proteins were separated in SDS-PAGE gels (10%), and transferred to polyvinylidene fluoride membranes (Merck KGaA, Darmstadt, Germany). Rabbit-anti-rat primary antibodies were used to bind target proteins including bax (Wanlei Biotechnology Co. Ltd, Shenyang, China, Catalogue No.: WL01637), bcl2 (Wanlei Biotechnology Co. Ltd, Shenyang, China, Catalogue No.: WL01556), GLP-1R (Bioss, Beijing, China, Catalogue No.: bs-1559R), and PPAR-γ (Santa Cruz Biotechnology, Inc., Dallas, TX, USA, Catalogue No.: sc-390740) at 4° C overnight. Following, HRP conjugated goat-anti-rabbit secondary antibody (Wanlei Biotechnology Co. Ltd, Shenyang, China, Catalogue No.: WLA023a) incubation administration was performed to detect primary antibodies. Revelation of proteins realized by ECL kit (Wanlei Biotechnology Co. Ltd, Shenyang, China, Catalogue No.: WLA006a).

### Chromatin immunoprecipitation (ChIP)

The ChIP assay was conducted according to the protocol from the manufacturer (Wanlei biotechnology co. Ltd, Shenyang, China, Catalogue No.: WLA122). PC12 cells were cross-linked by 1% formaldehyde (10 min at room temperature) before ultrasonic splintering. Chromatin solutions were incubated with 4 μg of anti-PPAR-γ antibody or with IgG, and rotated overnight at 4° C. Complexes were collected with protein A Sepharose beads for 1 hour at 4° C. To purify the immunoprecipitated DNA, beads were treated with DNase-free RNase A and proteinase K. And then, DNA was resuspended by distilled water. To amplify the GLP-1R promoter regions containing PPAR-γ, 5′-CAAGTCCACGCTGACACTC-3′ and 5′-GCTCTGTAAACAGCTTGATGAA-3′ were used as forward and reverse primers respectively [41]. After amplification, PCR products were analyzed on a 2% agarose gel. For quantification of the ChIP assay, input genomic DNA and immunoprecipitated DNA were amplified by real-time PCR.

### Plasmatic concentration of GLP-1 and CML measurements

All rats were anesthetized by 4% halothane anesthesia and sacrificed after the cognitive function tests. Fasting blood samples and fresh brain tissue were collected for further research. Blood samples (50μl) were collected from rats’ angular veins after fasting for 8 hours. Plasmatic GLP-1 (CUSABIO, Wuhan, China, Catalogue No.: CSB-E08117r) and CML (LifeSpan BioSciences, Seattle, WA, USA, Catalogue No.: LS-F27924) levels were measured by ELISA according to the Manufacturer's instructions.

### Immunofluorescence

Fresh brains of rats were isolated and embedded by OTC (Sakura Finetek Japan Co., Ltd, Tokyo, Japan), and then, they were cut to get frozen 10μm sections. After slices were repaired in the high-pressure cooker by citrate antigen retrieval solution (Beyotime, Shanghhai, China, Catalogue No.: P0081) and blocked by 5% normal goat serum (Beyotime, Shanghai, China, Catalogue No.: C0265) (1 hour at room temperature). They were incubated with rabbit-anti-rat primary antibody to GLP-1R (Santa Cruz Biotechnology, Inc., Dallas, TX, USA, Catalogue No.: sc-390774) and PPAR-γ (Santa Cruz Biotechnology, Inc., Dallas, TX, USA, Catalogue No.: sc-390740) at 4° C overnight. After washing for 3 times, Alexa Fluor 594 conjugated goat-anti-rabbit secondary antibody (Proteintech, Wuhan, China, Catalogue No.: SA00013-3) was used to detect the primary antibody. Finally, sections were stained by DAPI (Beyotime, Shanghhai, China, Catalogue No.: P0131) and washed for 3 times with PBS. GLP-1R (PPAR-γ) and DAPI (in hippocampal dentate gyrus and cortex) were observed by a fluorescence microscope (Olympus, Japan) after triggered at 594 nm and 358 nm respectively. Image were captured by (CellSens Standard).

### Statistical analysis

All data were described as mean ± standard deviation. Statistical differences were determined by using Student’s t-tests, and one-way ANOVA followed by LSD for multiple-comparison tests. Data were analysis by SPSS 22.0 (SPSS Inc., Chicago, IL, USA). P<0.05 was considered as significant difference.

## RESULTS

### CML induced PC12 cells apoptosis

**Figure 1:**
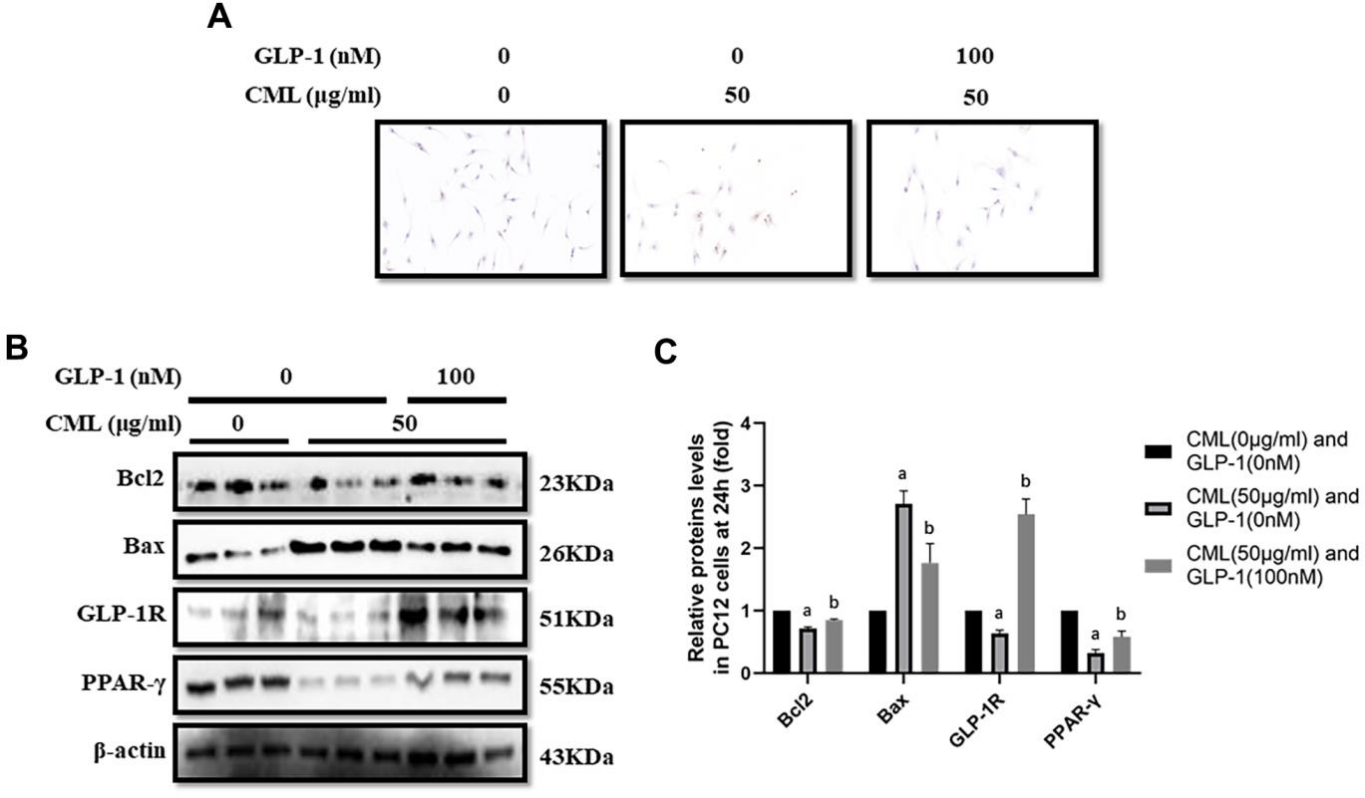
**GLP-1 attenuated apoptosis of PC12 cells induced by CML.** Results in (**A**) showed that GLP-1 restored the PC12 cells apoptosis induced by CML. (**B**) Showed the western blotting results of bcl2, bax, GLP-1R and PPAR-γ. “a” in (**C**) showed the down-regulated bcl2, PPAR-γ and GLP-1R as well as up-regulated bax in PC12 cells with 50 μg/ml CML, compared with those without CML (all *P<0.05*). “b” in (**C**) showed up-regulated bcl2, PPAR-γ and GLP-1R as well as down-regulated bax in PC12 cells with 50 μg/ml CML and 100 nM/ml GLP-1, compared with those with 50 μg/ml CML but without GLP-1 (all *P<0.05*). Data are represented as mean ± SD; n = 3 per group for results of western blotting.

To investigate the effect of CML on cells viability, CML (0, 12.5, 25, 50, 75 and 100 μg/ml) was used to treat PC12 cells for 6, 12, and 24 hours (supplementary Figure 1A–1C). We found significant cells viability decline at 24 hours in the medium with 50, 75, and 100 μg/ml CML (Supplementary Figure 1C). And then, we detected the levels of apoptosis by TUNEL assay. Indeed, increased apoptosis in PC12 cells was observed with 50 μg/ml CML, compared with that without CML (Figure 1A). Additionally, Compared with PC12 cells in medium without CML, we measured down-regulated bcl2 level and up-regulated bax level in PC12 cells with CML (50 μg/ml) (Figure 1B, 1C).

### CML down-regulated GLP-1R and PPAR-γ levels in PC12 cells

GLP-1R and PPAR-γ levels were also measured in PC12 cells stimulated by CML. GLP-1R and PPAR-γ levels were significantly down-regulated by CML with a concentration of 50 μg/ml (Figure 1B, 1C).

### GLP-1 treatment restored the apoptosis and up-regulated PPAR-γ levels

CML (50μg/ml) medicated PC12 cells viability declines were restored by GLP-1 at concentrations of 100nM, 200nM, and 500 nM in PC12 cells (Supplementary Figure 1D). In addition, decreased apoptosis was observed in PC12 cells treated with GLP-1 by TUNEL assay (Figure 1A). Furthermore, we showed up-regulation of bcl2 and down-regulation of bax in PC12 cells treat with GLP-1 at a concentration of 100nM for 24 hours (Figure 1B, 1C). To further explore the mechanism of the protecting effect of GLP-1 from apoptosis in neurons, levels of PPAR-γ and GLP-1R were measured. Higher PPAR-γ and GLP-1R levels were measured in CML treated PC12 cells with GLP-1 than that without GLP-1 (Figure 1B, 1C).

### Inhibition of PPAR-γ abolished the protective effect of GLP-1 and impaired the expression of GLP-1R

**Figure 2:**
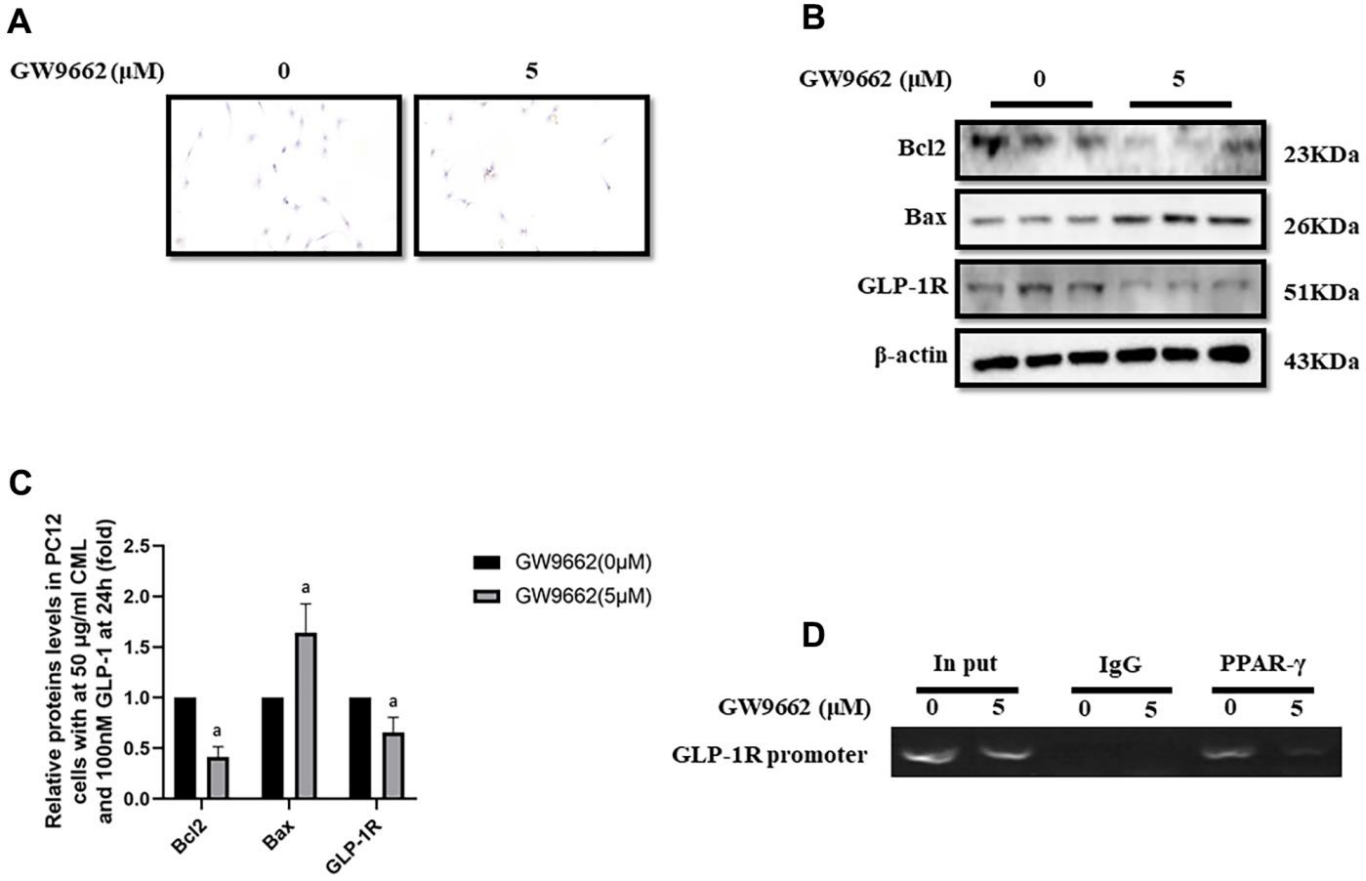
**PPAR-γ inhibition abolished the protective effect of GLP-1 on PC12 cells from apoptosis induced by CML.** Results in (**A**) showed GW9662 abolished the protective effect of GLP-1 on PC12 cells from apoptosis induced by CML. (**B**) Showed the western blotting results of bcl2, bax, and GLP-1R. “a” in (**C**) showed the down-regulated bcl2 and GLP-1R as well as up-regulated bax levels between PC12 cells (treated by 50ug/ml CML and 100 nM GLP-1) with and without 5uM GW9662. Results in (**D**) showed the direct interactivity between PPAR-γ and GLP-1R promoter sequence. Data are represented as mean ± SD; n = 3 per group for results of western blotting.

To clarify the important role of PPAR-γ involved in the process of GLP-1 alleviated PC12 apoptosis, GW9662, a selective inhibitor of PPAR-γ, was used to block the function of PPAR-γ. Interestingly, GW9662 (5, 10, 20, and 50 μM) significantly decreased the viability of PC12 cells with 50 μg/ml and 100nM GLP-1 at 24 hours (Supplementary Figure 1E). In addition, 5 μM GW9662 abolished the protective effect of GLP-1 from CML induced apoptosis detected by TUNEL assay (Figure 2A) or showed by bax or bcl2 levels (Figure 2B, 2C). Furthermore, the blocking of PPAR-γ also decreased the levels of GLP-1R in PC12 cells (Figure 2B, 2C). To investigate the association between GLP-1R and PPAR-γ, ChIP assay was conducted. Surprisingly, we detected the promoter sequence of GLP-1R in the mixture pulled down by PPAR-γ antibody (Figure 2D).

### Diabetic rats showed cognitive dysfunction

**Figure 3:**
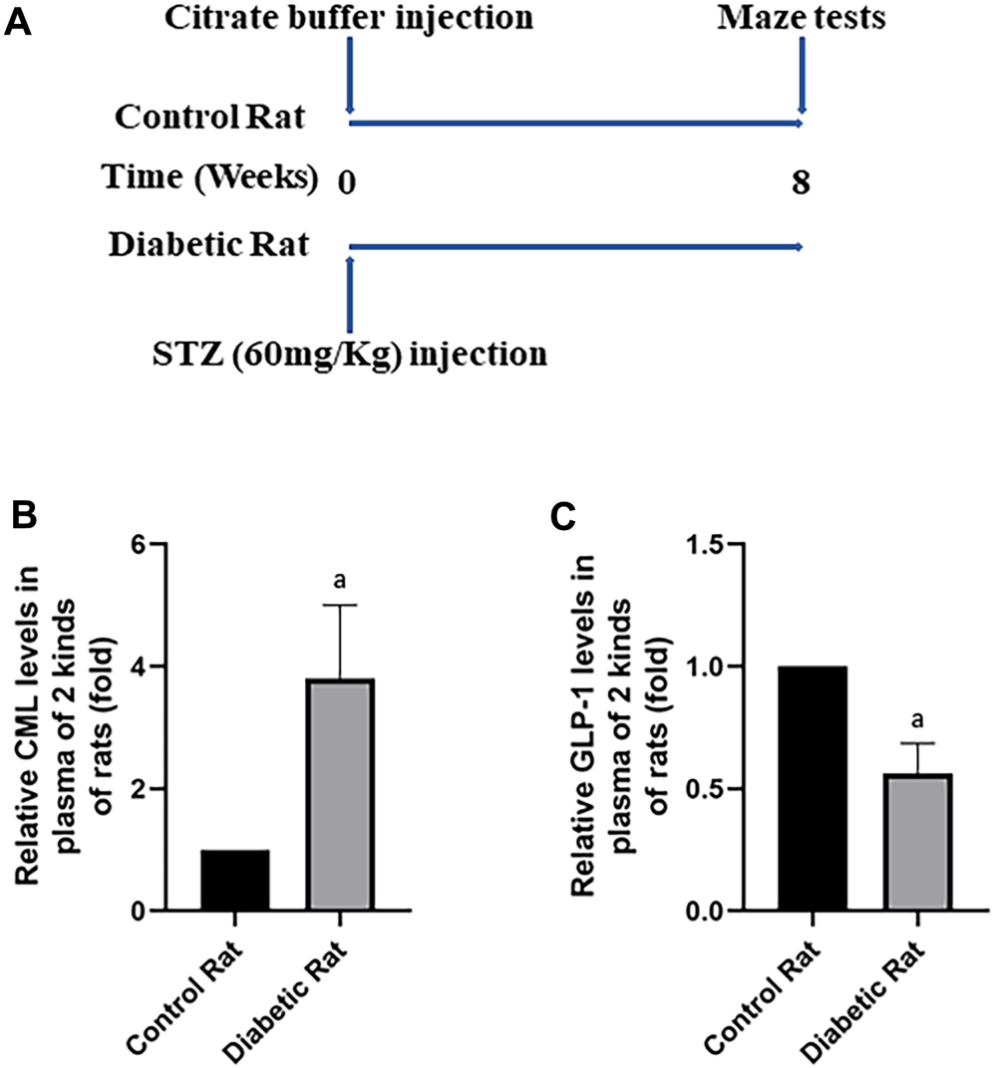
**Diabetic rats showed increased CML and decreased GLP-1 levels in plasma.** In (**A**), we showed treatments of Wistar rats. Each rat was given a large doze of STZ or citrate buffer (PH 4.5) to get diabetic rat or control rat. After 8 weeks, cognitive functions of all rats were tested by water maze. “a” in (**B**) showed increased CML levels, while in (**C**) showed decreased GLP-1 levels in the plasma of diabetic rats, compared with control rats. Data are represented as mean ± SD; n=4 per group for results of ELISA.

To confirm the cognitive function impairment in diabetes, Wistar rats (aged 6 weeks) were injected with a large doze of STZ. After 8 weeks, Morris water maze tests were performed to compare the cognitive function of rats with and without diabetes (Figure 3A). The path of rats found the target platform was showed in (Supplementary Figure 2A). Indeed, compare to control rats without diabetes, diabetic rats showed longer escape latency and path length after 5 days training (Supplementary Figure 2B, 2C). Furthermore, decreased percentage of time spend in the target quadrant and frequency of crossing platform area were recorded in rat with diabetes (Supplementary Figure 2D, 2E).

### Presence of increased CML and decreased GLP-1 in diabetic rats

**Figure 4:**
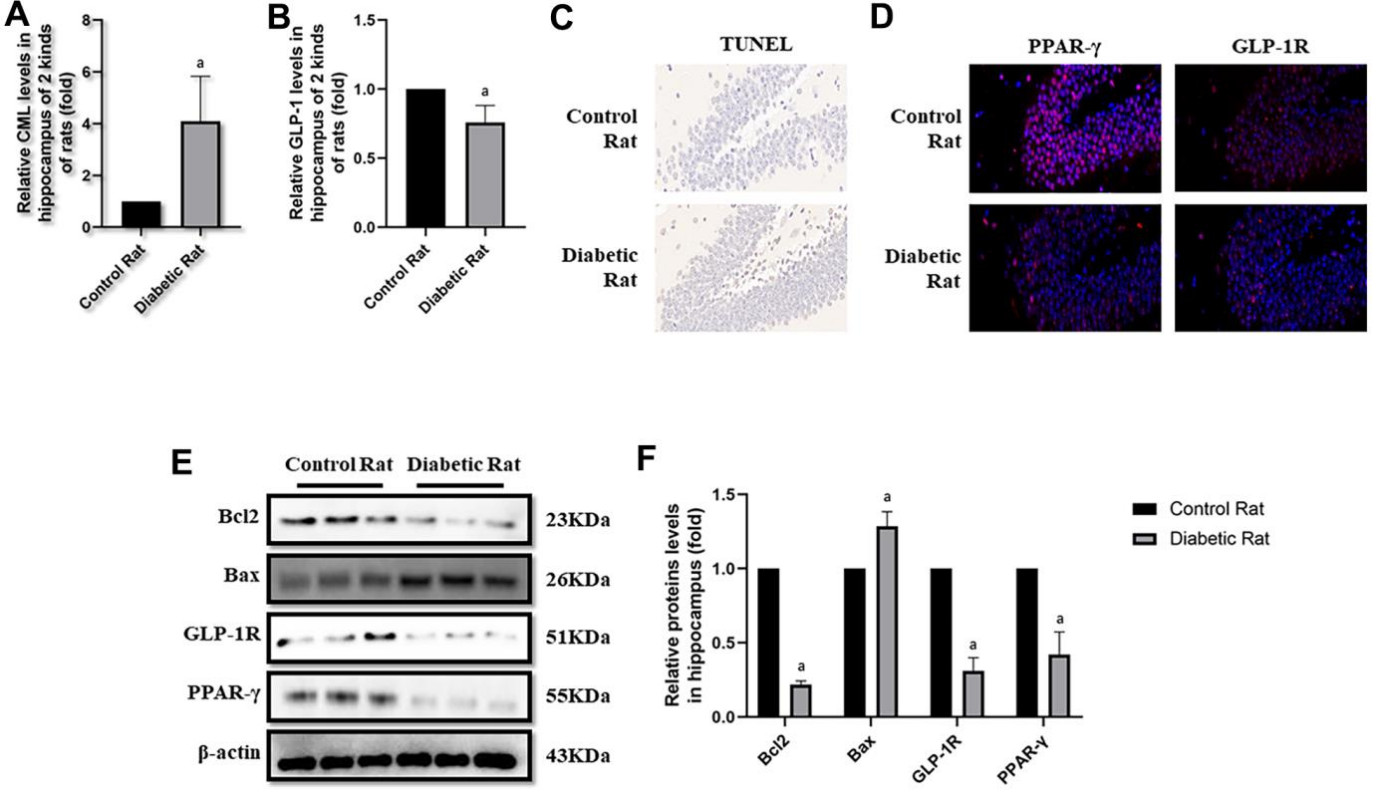
**Levels of apoptosis were elevated in hippocampus of diabetic rats.** “a” in (**A**) showed elevated CML levels in hippocampus of diabetic rats, compared with control rats (*P<0.05*). “a” in (**B**) showed decreased GLP-1 levels in hippocampus of diabetic rats, compared with control rats (*P<0.05*). Results in (**C**) showed increased apoptosis in hippocampus of diabetic rats, compared with control rats. Results in (**D**) showed down-regulated PPAR-γ and GLP-1R in hippocampus of diabetic rats, compared with control rats. (**E**) Showed the western blotting results of bcl2, bax, GLP-1R and PPAR-γ. “a” of (**F**) showed down-regulated bcl2, GLP-1R and PPAR-γ levels and up-regulated bax levels in hippocampus of diabetic rats, compared with control rats (all *P<0.05*). Data are represented as mean ± SD; n=4 per group for results of ELISA. n=3 per group for results of western blotting.

**Figure 5:**
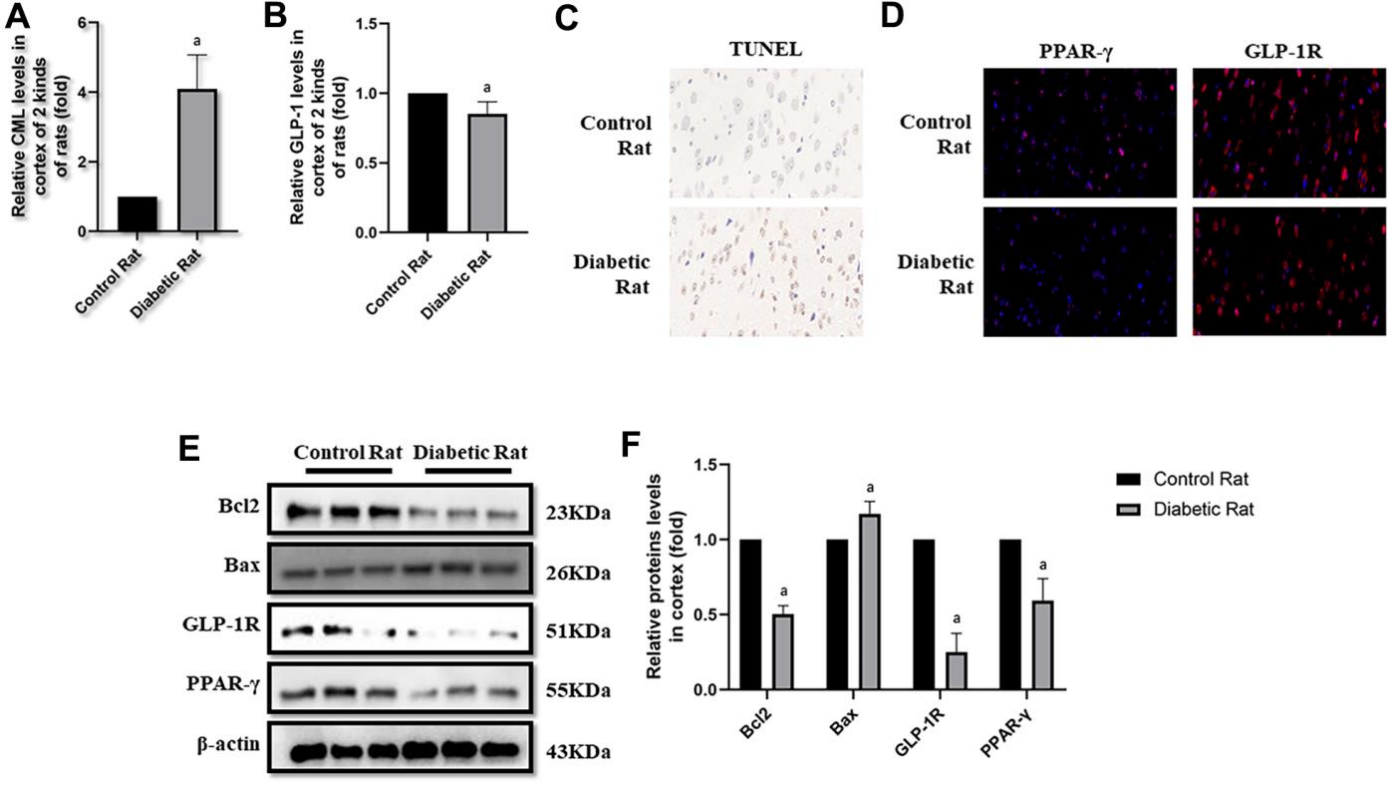
**Levels of cells apoptosis were elevated in cortex of diabetic rats.** “a” in (**A**) showed elevated CML levels in cortex of diabetic rats, compared with control rats (*P<0.05*). “a” in (**B**) showed decreased GLP-1 levels in cortex of diabetic rats, compared with control rats (*P<0.05*). Results in (**C**) showed increased apoptosis in cortex of diabetic rats, compared with control rats. Results in (**D**) showed down-regulated PPAR-γ and GLP-1R in cortex of diabetic rats, compared with control rats. (**E**) Showed the western blotting results of bcl2, bax, GLP-1R and PPAR-γ. “a” of (**F**) showed down-regulated bcl2, GLP-1R and PPAR-γ levels and up-regulated bax levels in cortex of diabetic rats, compared with control rats (all *P<0.05*). Data are represented as mean ± SD; n=4 per group for results of ELISA. n=3 per group for results of western blotting.

After the tests of cognitive function, plasmatic CML and GLP-1 levels were measured to explore the reasons of diabetic cognition decline. CML levels elevated more than 4 times, while GLP-1 levels decreased nearly half (Figure 3B, 3C) in diabetic rats. To further explore the reason of diabetic cognition decline, the sizes of brains were observed (Supplementary Figure 2F). However, we did not find any difference in brain sizes between control rats and diabetic rats. While increased CML, decreased GLP-1 levels in hippocampus and cortex were measured by ELISA (Figure 4A, 4B as well as Figure 5A, 5B).

### Diabetic rats showed more cells apoptosis as well as decreased GLP-1R and PPAR-γ in brain

Here, we observed the apoptosis in hippocampus and cortex by TUNEL assay. Interestingly, we not only detected more apoptosis in hippocampus but also in cortex tissue in diabetic rats than control rats (Figures 4C, 5C). Additionally, bcl2 and bax levels were measured to confirm the apoptosis. Indeed, elevated bax levels were discovered in diabetic rats, compared to rats without diabetes. Moreover, bcl2 levels are higher in control rats than rats with diabetes (Figure 4E, 4F as well as Figure 5E, 5F). The above results showed impaired GLP-1 levels in plasma and brain as well as neuronal apoptosis of diabetic rats. Therefore, GLP-1R levels in hippocampus and cortex were also measured. Down-regulated GLP-1R levels were found in these two parts of diabetic cerebrum by immunofluorescence and western blotting. Furthermore, PPAR-γ levels were also down-regulated detected by immunofluorescence and western blotting (Figure 4D–4F as well as Figure 5D–5F).

## DISCUSSION

AD is a huge challenge [5] worldwide, with limited efficient therapy. This pathology processes with three phases, (a) a preclinical period, beginning with asymptomatic accumulation of Aβ, leading to early neurodegeneration and then to subtle cognitive symptoms [42, 43], (b) a prodromal period, MCI [44, 45]; and (c) dementia due to AD [46]. T2DM is associated with MCI [47], and a risk factor for MCI progressing to dementia due to AD [48]. Neuronal apoptosis runs through these three stages. Hyperglycemia is the most important risk factor of cognitive decline in T2DM patients and promotes the conversion of MCI patients to those with AD. AGEs are formed in the condition of hyperglycemia in diabetes and associated with diabetic complications [49]. CML is a certain component of AGEs [33]. So, CML was used to induce the apoptosis of PC12 cells. Indeed, PC12 cells apoptosis levels were elevated by CML. GLP-1 and its analogue, showed their neuroprotective effect in mice [23, 24] and human [50] via binding to GLP-1R.

So, we guess that GLP-1 could regulate CML induced damage, including apoptosis in PC12 cells by binding to GLP-1R. To verify our hypothesis, GLP-1 treatment administrations were performed. We demonstrated more apoptotic cells in the medium with CML than that the medium without CML. Results in this present study are consistent with the research of Chen et al, showed AGEs activated apoptosis in SH-SY5Y cells [12]. Interestingly, we found a decreased GLP-1R levels in PC12 cells stimulated by CML. Similarly, GLP-1R defective mice showed declined cognition [25], and restored by GLP-1R over-expression [51]. PPAR-γ, an important receptor associated with apoptosis and proliferation in several kinds of cells [29, 52–54], including neurons [55, 56], is also regulated by AGEs in diabetes [30, 57]. In this work, PPAR-γ levels were down-regulated by CML. Additionally, cellular apoptosis triggered by CML was alleviated by GLP-1. This indicated the neuroprotective effect of GLP-1. Indeed, liraglutide and lixisenatide, analogues of GLP-1, could cross the BBB to play a role of neuroprotection [58, 59]. Although most GLP-1 was secreted by intestinal L cells [14], and inactivated by DPP-4 within 2 minutes [60], several cells in the brain could also produce an amount of GLP-1 [19, 20]. Moreover, GLP-1 significantly increased levels of PPAR-γ and GLP-1R down-regulated by CML.

Although GLP-1R and PPAR-γ play their important role in apoptosis, the interaction between them in CML induced apoptosis remains uncertain. To further determine the role of PPAR-γ in GLP-1 alleviated neuronal apoptosis, PPAR-γ blocking assay was performed in PC12 cells damaged by CML and remedied by GLP-1. Not surprisingly, GW9662, a specific blocker of PPAR-γ, abolished the protective effect of GLP-1 on PC12 cells from CML induced apoptosis. Additionally, the promoter sequence of GLP-1R was detected in the mixture pulled down by PPAR-γ antibody. These above results showed that GLP-1R expression may be regulated PPAR-γ located in nuclear via binding to the promoter of GLP-1R.

In order to confirm the protective effect of GLP-1 on neuronal apoptosis found *in vitro*, diabetic rats induced by STZ were used *in vivo*. Additionally, water maze was used to test their cognitive function. Indeed, we found significant cognitive dysfunction in diabetic rat. To confirm the effects of CML and GLP-1 in animal study, their levels were measured in plasma and brain of diabetic rats. Interestingly, we not only detected increased CML and decreased GLP-1 levels, but also observed elevated cells apoptosis levels in diabetic rats. These results determined that GLP-1 may involve in CML triggered cells apoptosis in the model of diabetic rats. To clarify the role of GLP-1 in apoptosis, GLP-1R was measured in hippocampus and cortex. We have already detected the interaction between PPAR-γ and GLP-1R promoter *in vitro*. In addition, PPAR-γ plays a promising role in neuroprotective effect may contribute to prevent apoptosis from AGEs [29, 30]. To further confirm the role of PPAR-γ in the neuroprotective effect of GLP-1, GLP-1R and PPAR-γ levels were also observed in the brain of rats with or without diabetes. Indeed, decreased GLP-1R and PPAR-γ levels were measured in the tissue of hippocampus and cortex. So, we suppose that GLP-1 may involve in neuronal apoptosis via PPAR-γ in diabetic rats with increased plasmatic CML levels.

**Figure 6:**
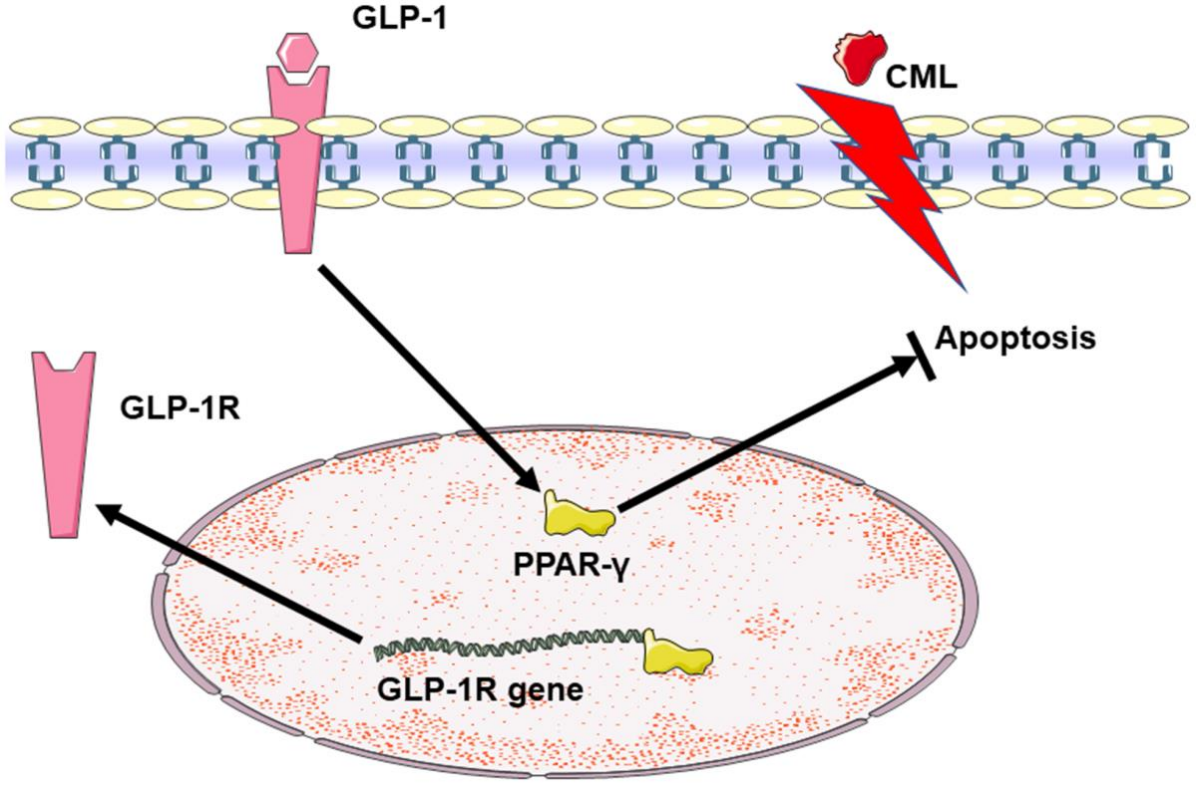
**Summary diagram of the relationship among GLP-1, CML and PPAR in cells apoptosis.** CML could induce the apoptosis of PC12 cells. GLP-1 could induce the expression of PPAR-γ by binding to GLP-1R. And then, PPAR-γ could attenuate the neuronal apoptosis. Additionally, PPAR-γ may promote the expression of GLP-1R by the interaction between PPAR-γ and the promoter sequence of GLP-1R.

In general, we demonstrated that GLP-1 may protect neurons from apoptosis in diabetes. Additionally, PPAR-γ is involved in this process (Figure 6). However, the mechanism involves in regulation of PPAR-γ by GLP-1 needs further exploration in the future research, especially for the animal experiments with GLP-1 analogues and PPAR-γ inhibitor. Despite these limitations, we still advocate bioactive GLP-1R agonists (Cannot be degraded by DPP4 inhibitors), was used for animal experiment, even for clinical patients with diabetic cognitive impairment or with higher risk of diabetic cognition decline.

## CONCLUSIONS

In conclusion, we indicated that GLP-1 may attenuate CML induced neuronal apoptosis via PPAR-γ, at least depends on PPAR-γ regulated GLP-1R expression partly. However, the mechanism of PPAR-γ regulated by GLP-1 remains need further study in our future work.

## Supplementary Material

Supplementary Figures
