# Supplementary material for: Glucagon-like peptide-1 attenuated carboxymethyl lysine induced neuronal apoptosis via peroxisome proliferation activated receptor-γ
Source: Aging (Albany NY). 2021 Jul 29;13(14):19013–27. doi: 10.18632/aging.203351 (PMC8351674; doi:10.18632/aging.203351)
Supplement: Supplementary Figures [file aging-13-203351-s001.pdf]

SUPPLEMENTARY FIGURES

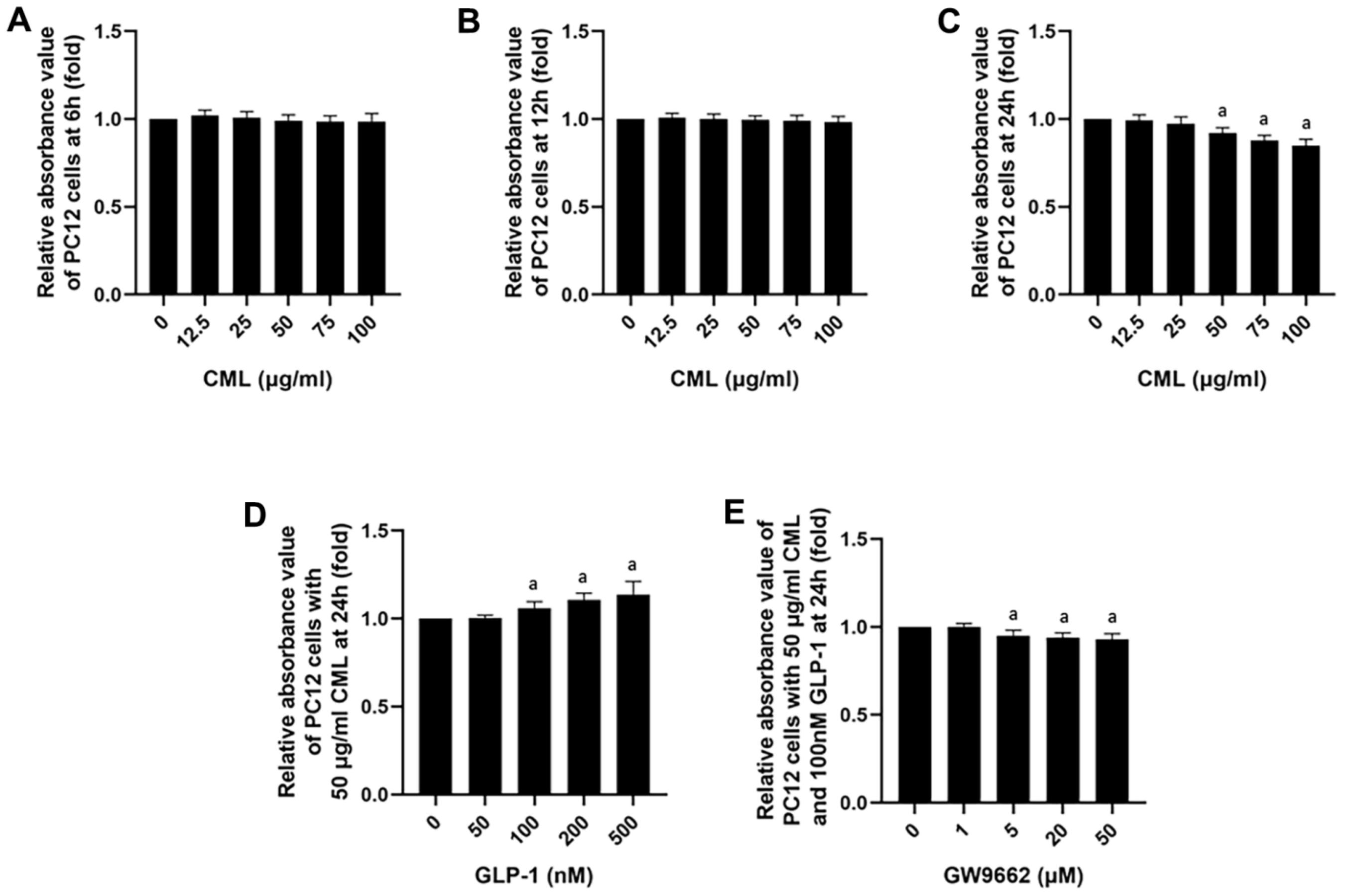

Supplementary Figure 1. PC12 cells viability with different treatments. Results showed in (**A**, **B**) did not show lower cells viability in PC12 cells with CML at than those without CML at 6h or 12h. "a" in (**C**) showed decreased cells viability in PC12 cells with CML (50, 75, or 100 μg/ml) at 24h, compared those without CML (all P<0.05). "a" in (**D**) showed higher cells viability of PC12 cells with 100, 200, or 500 nM/ml GLP-1 than those without GLP-1 (all P<0.05). "a" in (**E**) showed lower cells viability of PC12 cells (treated by 50ug/ml CML and 100 nM GLP-1) with 5, 10, 20, 50 uM GW9662 than those without GW9662 (all *P<0.05*).

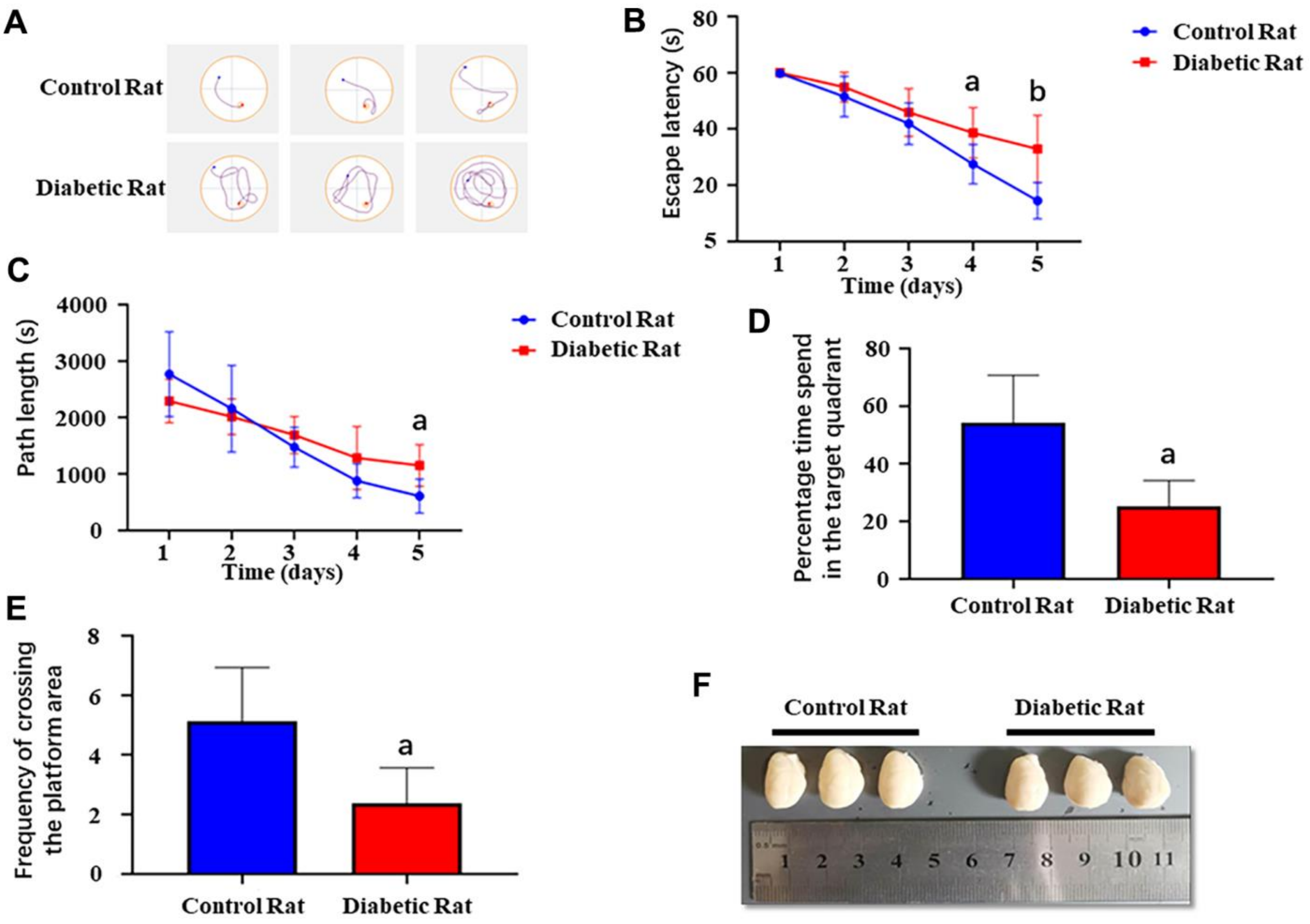

Supplementary Figure 2. Cognitive function was impaired in diabetic rats. In (**A**), we showed the swimming pathways of rats found the platform. "a" and "b" of (**B**) showed different escape latency at the 4th day and the 5th day respectively. "a" of (**C**) showed different path length at the 5th day. "a" of (**D**, **E**) showed different percentage of time spent in the target quadrant and frequency of crossing platform area between diabetic rats and control rats (all *P<0.05*). In (**F**), we did not find significant difference between brain sizes of diabetic rats and control rats. Data are represented as mean ± SD; n=8 per group for results of water maze.
